# ROLE OF INFLAMMATION AS SEX-SPECIFIC MEDIATOR BETWEEN CHRONIC STRESS AND COGNITIVE FUNCTION

**DOI:** 10.1093/geroni/igae098.3051

**Published:** 2024-12-31

**Authors:** Laura Laiton Jimenez, Idan Shalev, Martin Sliwinski, Yogasudha Veturi

**Affiliations:** The Pennsylvania State University, University Park, Pennsylvania, United States; The Pennsylvania State University, University Park, Pennsylvania, United States; Pennsylvania State University, University Park, Pennsylvania, United States; The Pennsylvania State University, University Park, Pennsylvania, United States

## Abstract

Long-term stress has been associated with poorer cognitive outcomes and increased risk of dementia, especially among women. Chronic stress is also known to lead to inflammation, whose role in neurodegeneration and associated comorbidities affecting immune function is well-established. Our investigation probes the mediating role of circulating inflammatory biomarkers (IB) in the social isolation stress (SIS) to cognitive function (CF) pathway, incorporating sex and age as moderating factors. Leveraging the MIDUS Refresher 1 cohort (N=3,800), we analyzed the role of SIS (frequency of social interactions with friends) on five CF tasks (word list recall, category verbal fluency, backward digit span, 30-second counting, and task-switching) and two IBs (C-reactive protein and interleukin-6). We employed a three-step approach to model these relationships: (1) CF~SIS; (2) IB~SIS; (3) CF ~SIS+IB. Poisson and linear regressions were employed as appropriate. After Bonferroni correction, we identified SIS as a significant predictor for the last three CF measures (p-value~0.01), with a pronounced sex effect in the last two (p-value~0.001). SIS was a significant predictor for interleukin-6 (p-value=0.0238), with a significant sex effect for C-reactive protein (p-value=0. 0.0003). Importantly, in step (3), the SIS-CF association diminished with the inclusion of IL6, highlighting its role as a putative mediator in this pathway. The sex effect remained unchanged. Next, we aim to analyze additional stressors (e.g. financial stress, perceived discrimination), examine the role of immune-related gene expression as a mediator, as well as investigate the role of inflammation-related comorbidities in the stress-CF pathway from electronic health records.

